# Exploring Changes in Valued Action in the Presence of Chronic Debilitating Pain in Acceptance and Commitment Therapy for Youth – A Single-Subject Design Study

**DOI:** 10.3389/fpsyg.2016.01984

**Published:** 2016-12-26

**Authors:** Mike K. Kemani, Gunnar L. Olsson, Linda Holmström, Rikard K. Wicksell

**Affiliations:** ^1^Functional Unit Behavioral Medicine, Karolinska University HospitalStockholm, Sweden; ^2^Department of Clinical Neuroscience, Karolinska InstitutetStockholm, Sweden; ^3^Department of Physiology and Pharmacology, Karolinska InstitutetStockholm, Sweden; ^4^Department of Women’s and Children’s Health, Karolinska InstitutetStockholm, Sweden

**Keywords:** ACT, single-subject design, chronic pain, children, adolescents, change processes

## Abstract

**Objective:** The objective of the study was to improve the understanding of processes of change in Acceptance and Commitment Therapy for youth with chronic debilitating pain by exploring the relation between individual change patterns in pain intensity and valued activities.

**Method:** A single-subject design across three adolescents suffering from longstanding debilitating pain was utilized. Pain intensity and participation in valued activities were rated daily. Visual analysis of the graphed data was performed to evaluate the effects of the intervention, and the relationship between pain intensity and values-based activity.

**Results:** The graphed data illustrated that pain levels did not decrease from the baseline period to the follow-up period. In contrast, compared to baseline ratings values oriented behaviors increased from the start of treatment to the follow-up period.

**Conclusion**: Results illustrate that increases in values-based behavior may occur without corresponding decreases in pain, and warrant further research on change processes in ACT for youth suffering from chronic pain.

## Introduction

A substantial number of children and adolescents suffer from longstanding pain, and previous studies report prevalence rates of 15–30% (El-Metwally et al., 2004). Among these a subset of persons suffer from pain related disability and reduced quality of life in addition to pain (Palermo, 2000; Miro et al., 2008; Hoftun et al., 2011). Importantly, several studies show that youth with longstanding pain enter adulthood with a substantial risk of chronicity (Walker et al., 1998; Brattberg, 2004).

Previous research provides empirical support for treatments based on a cognitive behavioral approach for pediatric longstanding pain (Eccleston et al., 2002; Hechler et al., 2015). Acceptance and Commitment Therapy (ACT) is a treatment within the cognitive behavioral field (Hayes et al., 1999). A number of studies illustrate the efficacy of ACT in improving pain related disability in adults with chronic pain (Hann and McCracken, 2014; Veehof et al., 2016), and a number of studies suggest the utility of ACT for children and adolescents with longstanding pain as well (Wicksell et al., 2007, 2009). The objective in ACT is to increase engagement in behavior that is in accordance with personal goals and values (i.e., approach behavior governed by appetitive motivating functions), also in the presence of pain and related distress, by promoting a willingness to experience interfering thoughts, sensations (e.g., pain), and emotions (Hayes et al., 2006).

A few clinical studies have illustrated the relevance of values-based behavior in improving pain related disability following ACT for adults with chronic pain (e.g., Vowles et al., 2014). Also, other researchers in the field of chronic pain argue that structured and detailed assessments of the patient’s personal overarching goals in important life domains would assist successful intervention (Schrooten et al., 2012). This underscores the relevance of clinical studies investigating values- and goal-based behavior. As a complement to clinical trials that use relatively few assessment points and group level data, single-subject studies that use frequent and idiographic assessments, of for example values-based action, may further enhance our knowledge of key processes of change in behavioral treatments for longstanding pain. This may in turn facilitate further development and improvement of treatment (Kazdin, 2009).

Thus, the present study aimed to explore the relationship between pain intensity and individualized assessments pertaining to pain related disability following ACT for youth with longstanding pain. This was done using a single-subject design including frequent assessments, before, during and after treatment, of pain intensity and values-based activities, that is, short- and long-term personally chosen behavioral goals in line with personal values.

## Method

### Design

A concurrent multiple baseline design across individuals was utilized (Kazdin, 1982), comprising a baseline (A), two intervention phases (B1 and B2) and a follow-up phase (C) replicated over three individuals. We randomized the order in which treatment was initiated for the patients (S1, S2, and S3). Baseline lengths of 12, 26, and 33 days were determined by taking into account the need for stable patterns in the assessment of pain intensity and valued activities (e.g., school attendance), as well as clinical considerations. One psychologist, a pain physician and a physiotherapist delivered the treatment. The psychologists and the pain physician had formal training in ACT, and all had clinical experience of using ACT with children and adolescents suffering from longstanding pain.

### Recruitment

Three adolescents (S1, S2, and S3), two 14 year olds and one that was 18 years of age, with longstanding pain (i.e., a pain duration of more than 3 months) were included in the study. The patients were referred from county councils outside the Stockholm area to the Behavioral Medicine Pain Treatment Services (BMPTS), at the Karolinska University Hospital. Initial medical and psychological assessments at the clinic were conducted during 2–3 days (6–8 sessions). The medical assessment was based on a semi-structured interview focusing on the medical history of each patient. At this assessment pain intensity was rated using numeric scales ranging from 0–10 to 0–100 with the endpoints “no pain at all” to “worst pain imaginable.” The psychological screening assessed the negative consequences of pain on different life domains based on a semi-structured interview, which also included clinical behavior analyses of relevant target behaviors. Also, valued activities (treatment goals) were defined for future assessment. Written consent to participate in the study was provided by both adolescents and parents and the Ethical Review Board in Stockholm approved the study.

### Assessment

Activities deemed personally important by the participants were collaboratively formulated and rated individually on a daily basis. S1 rated the “Number of classes I attended today, of the total number of classes” (e.g., 4/5). S2 rated the “Number of minutes I bowled today” and the “Number of meters I jogged today.” S3 rated the “Number of minutes I walked without support today,” and the “Number of minutes I played tennis today.” In addition, the item “How much pain have you experienced today,” was rated daily on an 11-point numerical scale ranging from “no pain at all” (0) to “worst pain imaginable” (10). The participants were instructed to perform the ratings at the end of each day and parents were instructed to assist and ensure that the ratings were performed according to instructions. Pain was rated from baseline (A) to 7–14 days past follow-up (C).

Additionally, data was collected by the child version of the Functional Disability Inventory (FDI) (Walker and Greene, 1991). This version of the FDI measures the impact of sickness on physical and psychosocial functioning, and consists of 15 questions measuring ambulation; social interaction; ability to perform household tasks; ability to eat, sleep and rest, attend school; and mobility. The FDI was administered three times, at treatment start (B1), at the end of treatment (B2), and at follow-up (C), approximately two months following the end of treatment.

### Data Analytic Approach

In single-subject designs the baseline data illustrates the trajectory of the variables over time under conditions that do not change (Boersma et al., 2004). And, if a change in the trajectory of a dependent variable occurs systematically following the intervention it increases the likelihood that the change is an effect of the intervention (Kazdin, 1982). Because each subject acts as his/her own control condition, and frequent assessments are made, these studies typically include a small number of participants. Multiple baselines across subjects increases the internal validity, and effects across subjects considerably builds a case for generality (Kazdin, 1982).

Visual non-statistical analyses of the graphed data within and between subjects were performed to evaluate if changes in the dependent variables (pain intensity and values-based behaviors, e.g., school attendance) were a consequence of treatment (Kazdin, 1982). More specifically, we evaluated the means and the variability of the ratings across phases. Substantial changes in these regards (e.g., the range), after the introduction of treatment, were indicative of a treatment effect. We also analyzed the level, or degree, of change between phases and considered a shift or rupture in the trajectory, that is, a considerable drop or increase in the ratings, following the onset of treatment an expression of a treatment effect. Additionally, we took into account the latency of change, in other words, when in time a change in the slope occurred. The closer in proximity to treatment introduction that change occurred, the more likely we deemed this change to be an effect of treatment. Microsoft Excel 2010 was used to graph the data and to calculate means (M) and range (R).

### Patient Characteristics

All patients lived with both parents. In addition to pain, two patients presented with psychiatric (S1) and somatic (S3) concurrent symptoms. Patient characteristics based on the initial clinical medical and psychological assessments are presented below. Previous medical investigations and treatments for the three patients are presented as Supplementary Material.

#### S1

S1 was a 14-year-old boy whose pain onset followed multiple minor foot injuries, such as sprains, at age 3. Over time pain gradually became more generalized and increased in intensity. At assessment S1 presented with generalized continuous spontaneous pain in his head, shoulders, back, knees, groin, and ankles, as well as recurrent pain in arms and wrists. He experienced his headache as the most disturbing. In addition, he reported that pain was triggered by brushing and touching of the skin, as well as by applying light pressure to the skin (i.e., mechanical and dynamic mechanical allodynia) of the shoulder area. Pain increased during and after physical activity, primarily in his feet and groin. Also, following physical activity he sometimes experienced a temporary brief loss of motor functioning in his legs. At the initial assessment S1 reported a current pain experience of 98 on a scale ranging from 0 (“no pain”) to 100 (“worst pain imaginable”). Using the same scale, he reported that his pain was 100/100 when at its highest and 70/100 at its lowest.

Prior to assessment at the BMPTS, he was diagnosed with social phobia and Asperger’s syndrome. S1 was also taking prescribed medication for anxiety and depression. S1 had been bullied in school during a period in the seventh grade. At assessment he attended the eighth grade and the bullying had ceased. A high level of pain related school absence was reported, and S1 was completely absent from school the past semester due to pain. He had stopped playing soccer and only sporadically played floorball (a type of field hockey), due to pain and social difficulties on the team.

#### S2

For S2, an 18-year-old male, pain debuted when he was 14 and the onset of pain could not be associated with any trauma or infection. Over time pain gradually generalized and increased in intensity, and at assessment S2 presented with continuous spontaneous back pain and mechanical dynamic allodynia in his back. He also experienced occasional shoulder and knee pain, especially during certain twisting movements of the knee. Walking was terminated after about 10 min due to pain. Pain was most intense in the mornings, and increased during physical activity. At assessment, S2 reported that his current pain intensity corresponded to a rating of 8.5 on scale ranging from 0 (“no pain”) to 10 (“worst pain imaginable”). His pain corresponded to a 10 when it was at its highest and a 6 when it was at its lowest.

Also, he presented with recurrent muscle spasms, fatigue and widespread loss of muscle tonus that resulted in a temporary inability to stand up. S2 attended the 3rd year of high school and had only been absent a few days due to pain the past semester. He had not gone bowling or played soccer in several years, due to pain.

#### S3

Pain onset for S3, a 14-year-old girl occurred at age 13. This happened approximately 4 weeks after an ovarian torsion surgery, and was triggered by a strain in the groin during tennis play. Following a medical procedure at another university hospital, in which a tube with a camera was inserted through the urethra into the bladder (i.e., a cystoscopy) during epidural anesthesia (a regional anesthesia injected into the back), S3 lost all sensory and motor functioning in her legs. S3 presented with continuous spontaneous pain in the genital area and left groin as well as severe pain triggered by pressure to, or touch of, the skin (i.e., allodynia) in the left groin. She also experienced pain from the lower left abdomen and the center of her back. Pain intensity increased during and following physical activity.

S3 attended eighth grade and had a high level of school absence, and was completely absent from school the past semester. She had stopped playing floorball and tennis due to pain and loss of sensory and motor functioning in her legs. Key clinical characteristics for the three patients are presented in **Table 1**.

**Table 1:** Key patient characteristics for S1, S2 and S3 at the initial clinical assessment.

	Age	Sex	PainDur^a^	Prim. pain loc.(other pain loc.)	Concurrent symptoms	Diagnoses(secondary diagnoses)
S1	14	M	132	Head (wide-spread)	Recurrent loss of motor function in lower legs.	Unspecified generalized pain (Asperger’s syndrome; Social phobia).
S2	18	M	48	Back (head)	Muscle spasms, widespread temporary loss of muscle tonus	Unspecified generalized pain.
S3	14	F	12	Groin (lower abdomen, back)	Loss of motor and sensory function in both legs	Unspecified generalized pain (unspecified pain in other areas of the lower abdomen; hyperesthesia; painful micturition; and unspecified paralytic syndrome).

^a^*Months*.

### Treatment

The first treatment period (B1) consisted of 4 days, and was initiated directly following baseline. For all patients, sessions with a physician, psychologist and physiotherapist were included. All sessions promoted acceptance of pain and related distress as well as engagement in values-consistent behavior. During B1 the physician delivered 2–4 sessions; the physiotherapist one session; and the psychologist 7–15 sessions. The second treatment period (B2) also consisted of 4 days and was initiated 3–4 weeks after B1. During B2 the physician delivered 1–3 sessions; the physiotherapist one session; and the psychologist 5–7 sessions. Each session lasted 45–75 min. Three to 7 weeks following B2 there was a 1–2 days follow-up (C). The physician and the physiotherapist delivered one session each with the patient and the parents, and the psychologist 2–3 sessions.

#### First Treatment Period, B1

During initial assessment behavioral goals were operationalized based on the patients’ values, in relation to for example family, school, leisure time, physical activity, and friends. At the start of B1 these values and goals were further discussed, as a way to motivate behavior change, and as a means to potentially reinforce behavioral patterns and direct behavior over extended periods of time, also in the presence of other aversive experiences such as pain and related distress. In conjunction with these discussions the physician and the psychologist provided information regarding the differences between acute and chronic pain, the complex and many times unclear etiology of longstanding pain, the high prevalence of such pain, and the potential downsides of a prolonged and extended search for an underlying and treatable pathophysiology. These discussions served to initiate a shift from seeking symptom reduction to increasing values-based action, even in the presence of pain.

To further motivate a shift from pain reducing behaviors to values-oriented behaviors, the short- and long-term workability of previously used behavioral strategies characterized by avoidance of pain and related distress (e.g., staying home from school) were collaboratively evaluated. This evaluation illustrated that avoidance strategies had led to a decrease in valued activities over time, without any corresponding decrease in pain and related distress. It also illustrated the difficulty of avoiding pain and related discomfort, while at the same time living an active and meaningful life.

In order to facilitate engagement in values consistent activities the psychologist introduced defusion and acceptance as alternative strategies to manage pain and related distress. Metaphors and experiential exercises were frequently used to enhance and elucidate the points addressed during sessions. The latter part of B1 focused on values-based behavior activation and the use of defusion and acceptance strategies while engaging in valued activities, such as attending classes in school and bowling. To facilitate in-session *in vivo* exposure to pain-inducing or distressing activities the psychologist and physiotherapist used various forms of physical activities, such as walking or pool exercises, depending upon type of symptoms and individually defined values. For S3, most sessions also included a focus on improving motor functioning in her legs. To achieve this, minimizing wheelchair use was promoted as a general strategy. Additionally, floor mobilization exercises (e.g., creeping) and tilt board exercises were utilized throughout treatment.

#### Second Treatment Period, B2

The second treatment period (B2) focused on the implementation of ACT strategies in everyday life. When needed, previously formulated behavioral goals were discussed and refined, such as increasing the time spent in school. The interaction with friends, parents and other significant adults was also addressed. For example, we discussed how the youth wanted to be coached toward increased valued living, and how this could be communicated to parents or friends. At follow-up (C), strategies to handle setback and relapse were discussed with both the patient and parents.

#### Parental Support

Broadly, over both treatment periods parent sessions were focused on improving coaching behaviors. Initially, parents were taught operant principles (contingency management), and how these principles applied to their child’s values and goals. In addition, parental distress and ineffective coaching behaviors were discussed based on clinical behavior analysis of critical situations. Subsequently, alternative ways of dealing with parental distress to promote the child’s behavioral activation were discussed. For example, the parents were encouraged to be accepting of their own distressing thoughts and emotions related to their child’s pain, as a way to undermine the impact of these thoughts and feelings on effective coaching behaviors.

## Results

Notably, pain remained at similar levels throughout treatment for all patients. However, pain varied more for S1 compared to S2 and S3. Compared to baseline (*M* = 3.6/5), class attendance increased (*M* = 4.3/5) for S1 following B1. Shortly following B2, S1 attended five out of five classes for five consecutive weeks until the ratings were discontinued.

S2, did not bowl or jog during baseline, but shortly following B1 bowling increased in both duration (*M* = 60 min/week) and frequency (*M* = 1 occasion/week). Following B2, there was a continued increase in bowling, in both duration (*M* = 210 min/week) and frequency (*M* = 2.8 occasions/week). Also, jogging increased shortly following B1, in both distance (*M* = 383 m/week) and frequency (*M* = 2 occasions/week). This increase continued steadily following B2 (*M* = 1419 m/week; *M* = 2.4 occasions/week). After follow-up (C) there was a reduction in jogging (*M* = 760 min/week; *M* = 1.5 occasions/week).

**FIGURE 1:**
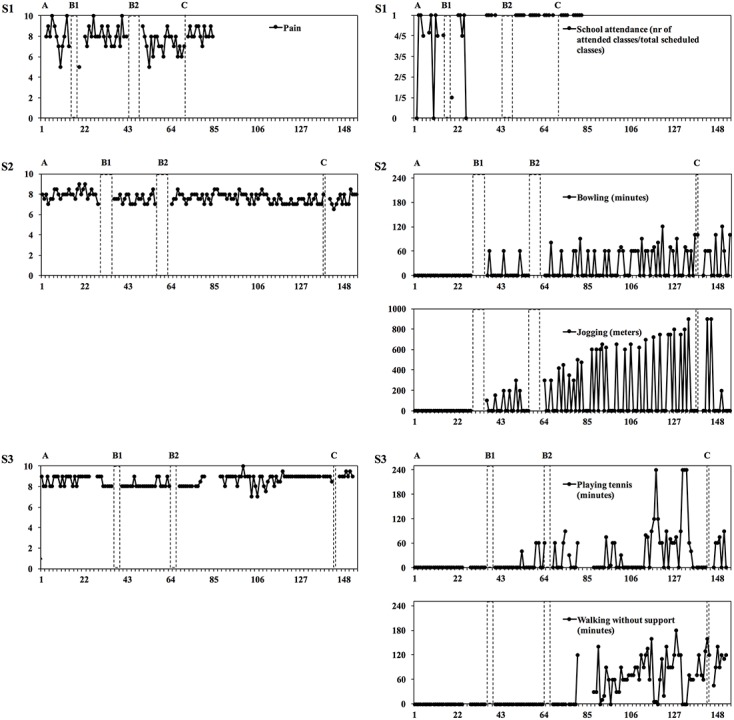
**Daily ratings of pain intensity and values-based behaviors are presented in graphs for S1, S2, and S3**.

S3 did not play tennis during baseline, but shortly following B1, there was an increase, both in duration (*M* = 40 min/week) and frequency (*M* = 0.75 occasions/week). Further increases shortly followed B2 (*M* = 241 min/week; *M* = 2.6 occasions/week). After follow-up (C) there was a decrease in playing tennis (*M* = 143 min/week; *M* = 1 occasion/week). S3 did not to walk without support during baseline, but this ability increased substantially following B2, in both duration (*M* = 241 min/week) and frequency (*M* = 2.6 occasions/week). There was a further increase in ability to walk without support (*M* = 593 min/week; *M* = 5.5 occasions/week) during the follow-up period. Means and ranges for the different variables, as well as the number of days for each phase, are presented in **Table 2** for each participant. Also, the individual daily assessments of the included variables are presented in graphs in **Figure 1**.

**Table 2:** Number of days for the respective phases, as well as means and ranges for the individual ratings, across all phases (A, B1, B2, and C) and participants (S1, S2, and S3).

		A		B1		B2		C	
	Variable	Nr	Mean (Range)	Nr	Mean (Range)	Nr	Mean (Range)	Nr	Mean (Range)
S1	Days^a^	12		13		20		13	
	Pain intensity^b^		8 (5)		8 (3)		7.3 (4)		8.3 (2)
	Class att. (att. classes/scheduled classes)		3.6/5 (0–4/5)		4.3/5 (0–5/5)		5/5		5/5
S2	Days	26		22		77		13	
	Pain intensity		8 (1)		7.5 (1)		7.6 (1)		7.5 (2)
	Bowling (minutes/week)		0		60 (60)		181 (330)		208 (380)
	Bowling (occasions/week)		0		1 (1)		2.8 (5)		2.9 (4)
	Jogging (meters/week)		0		383 (500)		1419 (2350)		760 (1800)
	Jogging (occasions/week)		0		2 (2)		2.4 (3)		1.5 (2)
S3	Days	33		23		74		6	
	Pain intensity		9 (2)		8 (1)		8.7 (2.5)		9 (0.5)
	Playing tennis (minutes/week)		0		40 (40)		241 (945)		143 (225)
	Playing tennis (occasions/week)		0		0.75 (2)		2 (6)		1 (1)
	Walking without support (minutes/week)		0		0		335 (600)		593 (605)
	Walking without support (occasions/week)		0		0		4.2 (7)		5.5 (6)

^a^*Phase length is reported as the number of days from the start of a specific phase (e.g., A) to the start of a new phase (e.g., B1). ^b^The item was rated from “No pain at all” (0) to “Worst pain imaginable.”*

The results from the assessments made of pain related functional disability at the end of treatment and at follow up 2 months after treatment, indicated that functional disability had decreased for the participants, especially for S2 and S3. Please see **Table 3** for specifc scores at the different time points for the three particpants.

**Table 3:** Functional Disability Inventory (FDI) scores for the three particpants (S1, S2, and S3).

	FDI pre^a^	FDI post	FDI 2mfu^b^
S1	29	26	-
S2	15	2	2
S3	50	27	36

*A dash represents missing data. ^a^Data collected at assessment. ^b^Data collected approximately 2 months after post-assessment.*

## Discussion

This study explored patterns of change in pain intensity and valued activities using daily assessments. Notably, pain reduction was not targeted in treatment, but is important to assess in order to evaluate the effects of treatment and the relationships between symptoms and improvements in disability. The pattern of results clearly suggests that changes in valued behaviors were independent of changes in pain intensity. The greatest increase in values oriented behaviors was seen following the second treatment phase (B2). The results align with results from previous studies on ACT for youth illustrating improvements in pain related disability (Wicksell et al., 2009). However, for adolescents the effect of ACT on pain intensity appears to vary across studies. In a study by Wicksell et al. (2009) results illustrated improvements in pain intensity following treatment, but in a study by Kanstrup et al. (2016) pain intensity was not reduced following treatment. Notably though, mediation analyses suggest that decreases in disability following ACT for adults as well as for youth are not primarily a function of pain reduction (Wicksell et al., 2010; Kemani et al., 2016), which the results from the current study also illustrate.

A number of methodological limitations should be noted. Limitations pertaining to the reliability of the visual analytic approach and to the generalizability of the results are of central concern. There is yet no clear consensus regarding the criteria for visual data analysis, particularly the interpretation of certain data patterns and how to establish the reliability of the effect (Deprospero and Cohen, 1979). Statistical methods have been suggested as a way to handle these problems (Kazdin, 2007), but it is yet unclear how statistical analyses should be conducted to be fully satisfactory given, for example, the usually small samples in these studies. Also, data collection relied heavily on self-report, which potentially undermines the reliability and validity of the results. In this regard, objective assessment, such as actigraphy, may complement self-ratings. Furthermore, the FDI was included mainly for comparisons with the daily ratings of values oriented behaviors. However, more frequent assessments using validated questionnaires that complement the individually formulated outcomes should be used, such as measures that assess emotional functioning and quality of life. Although treatment staff continuously discussed fidelity to treatment, adherence and therapist competence should be analyzed using recordings of the sessions and a standardized coding system.

More studies with larger samples are needed to determine the generalizability of the findings presented here. However, future studies should also consider the strengths of the current study, in essence, the focus on individual change in relation to personally important outcomes using multiple assessments during the course of the different phases related to treatment. Additionally, these studies should utilize designs with adequate experimental control that meet the requirements for adequate statistical analyses.

A number of studies on ACT for chronic pain have investigated the mediating role of core ACT processes, such as psychological inflexibility, in improving outcomes (Wicksell et al., 2010, 2013). However, only a few studies (Kemani et al., 2016) have modeled change more carefully using multiple assessments of the proposed process and outcome variables (e.g., acceptance and pain related disability), and evaluated the precedence of change in the process variable in relation to the outcome. These aspects need to be further studied and single-subject designs provide a framework to explore both the specificity of these process variables and the temporal precedence of change in these variables in relation to the outcome variables.

Clinically, repeated assessments of individualized outcomes can be used concurrently with validated questionnaires or other means of data collection (e.g., actigraphy) to provide detailed feedback as to the efficacy of treatment, and as a basis for discussing potential adjustments to the treatment in cases when desired change does not occur. In conclusion, results indicate that values-based activity can improve even when reductions of pain do not occur. The study also points to the importance to further research the effects of ACT for patients with complex symptoms, as well as the circumstances under which desired change occurs.

## Ethics Statement

The study was approved by the Ethical Review Board in Stockholm. The participants (and their parents) were informed that the data and results were going to be analyzed and that the results would be presented in a scientific publication, in such a way that they as individuals could not be identified. Thus, we have altered certain characteristics of the participants in order to ensure their anonymity.

## Author Contributions

MK, GO, and RW was involved in the design of the study, data preparation, visual analyses, and manuscript preparation. LH was involved in the data preparation, visual analyses and manuscript preparation.

## Conflict of Interest Statement

The authors declare that the research was conducted in the absence of any commercial or financial relationships that could be construed as a potential conflict of interest. The reviewer SM declared a past co-authorship with one of the authors RW to the handling Editor, who ensured that the process met the standards of a fair and objective review.
