# Supplementary material for: Exploring Changes in Valued Action in the Presence of Chronic Debilitating Pain in Acceptance and Commitment Therapy for Youth – A Single-Subject Design Study
Source: Front Psychol. 2016 Dec 26;7:1984. doi: 10.3389/fpsyg.2016.01984 (PMC5183655; doi:10.3389/fpsyg.2016.01984)
Supplement: Supplementary file 1 [file Presentation_1.pdf]

SUPPLEMENTARY MATERIAL

Patient characteristics

S1

A rheumatologic evaluation and a MRI of the brain identified no clear pathophysiology that could explain the pain. Furthermore, physical examination did not reveal any concerning medical reasons for the temporary brief loss of motor functioning in the legs following physical activity. Naproxen, paracetamol and codeine had limited effects on pain, and prednisolone (up to 60 mg) had a moderate effect on shoulder pain. Physiotherapeutic treatment had no clear effects, but acupuncture and Transcutaneous Electrical Nerve Stimulation (TENS) resulted in temporary pain relief.

S2

Investigations by pediatricians and rheumatologists identified no clear pathophysiology. Paracetamol and NSAIDs had been tested without effects on pain. Gabapentin (600 mg x 3) resulted in side effects (dizziness, tiredness and memory problems) without any effects on pain. Paracetamol was used for the headache, with moderate effects. Physiotherapy and strength training had an initial effect on functioning. TENS and acupuncture led to increased pain. Chiropractic and a naprapathic treatment, mainly comprising manual therapy and massage, did not have any effects on pain.

S3

Neurological investigations, including MRI, were performed without any pathological findings related to pain or the loss of sensory and motor functioning in the legs. Gabapentin (1800 mg/day) had an effect on pain, but side effects of blurred vision, and changes in mood and behavior were experienced. Due to these side effects, efforts to reduce dosage were made. However, pain increased when dosages were lowered, which complicated tapering.

Amitriptyline, tramadol and clonidine had no effect on pain, but resulted in dizziness and tiredness. S3 reported intolerance to morphine. She had no sensory functioning in her legs and was completely paretic in both legs, and thus used a wheel chair.

Treatment

Acceptance and Commitment Therapy

From an ACT perspective, avoidance of pain and related distress is conceptualized as the core behavioral problem that results in negative long-term outcomes, e.g. disability and reduced quality of life. The excessive, or inappropriate, impact of negative thoughts and emotions on behavior (denoted as cognitive fusion) is a key psychological process influencing avoidance behavior. In order to undermine the impact of these experiences on behavior the patient is encouraged to distance him/herself from pain and distress (defusion) and to willingly experience, negative thoughts, emotions and bodily sensations that cannot be directly changed (acceptance). Both defusion and acceptance are promoted when they serve engagement in activities that are meaningful over extended periods of time (values), although possibly painful or fear provoking. The treatment objective is to increase psychological flexibility, defined as the ability to act effectively in accordance with personal values, even in the presence of interfering thoughts, emotions, and bodily sensations.
